# Dining from the coast to the summit: Salmon and pine nuts determine the summer body condition of female brown bears on the Shiretoko Peninsula

**DOI:** 10.1002/ece3.7410

**Published:** 2021-03-18

**Authors:** Yuri Shirane, Mina Jimbo, Masami Yamanaka, Masanao Nakanishi, Fumihiko Mori, Tsuyoshi Ishinazaka, Mariko Sashika, Toshio Tsubota, Michito Shimozuru

**Affiliations:** ^1^ Graduate School of Veterinary Medicine Hokkaido University Sapporo Japan; ^2^ Shiretoko Nature Foundation Shari Japan

**Keywords:** body condition, brown bear, diet, nutritional status, reproductive status, *Ursus arctos*

## Abstract

Body condition in mammals fluctuates depending on energy intake and expenditure. For brown bears (*Ursus arctos*), high‐protein foods facilitate efficient mass gain, while lipids and carbohydrates play important roles in adjusting dietary protein content to optimal levels to maximize energy intake. On the Shiretoko Peninsula, Hokkaido, Japan, brown bears have seasonal access to high‐lipid pine nuts and high‐protein salmon. To assess seasonal and annual fluctuation in the body condition of adult female brown bears in relation to diet and reproductive status, we conducted a longitudinal study in a special wildlife protection area on the Shiretoko Peninsula during 2012–2018. First, analyses of 2,079 bear scats revealed that pine nuts accounted for 39.8% of energy intake in August and salmon accounted for 46.1% in September and that their consumption by bears varied annually. Second, we calculated the ratio of torso height to torso length as an index of body condition from 1,226 photographs of 12 adult females. Results indicated that body condition continued to decline until late August and started to increase in September when salmon consumption increased. In addition, body condition began to recover earlier in years when consumption of both pine nuts and salmon was high. Furthermore, females with offspring had poorer body condition than solitary females, in particular in late August in years with low salmon consumption. Our findings suggest that coastal and subalpine foods, which are unique to the Shiretoko Peninsula, determine the summer body condition of female brown bears, as well as their survival and reproductive success.

## Graphical abstract

To clarify seasonal and annual fluctuation in the body condition of adult female brown bears in relation to food habits and reproductive status, we conducted a 7‐year longitudinal study that included scat sampling and direct observation of bears in a special wildlife protection area on the Shiretoko Peninsula. Using photographic evaluation to assess the body condition of free‐ranging brown bears, we noninvasively monitored the body condition of identifiable bears throughout the study period. Our findings suggest that coastal and subalpine foods, which are unique to the Shiretoko Peninsula, determine the summer body condition of female brown bears as well as their survival and reproductive success.

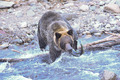

## 1 INTRODUCTION

A variety of mammal species experience fluctuations in body condition as a result of varying energy intake and expenditure (Boswell et al. 1994; Fietz & Ganzhorn, 1999; Parker et al. 2009). When highly nutritious food resources are available, individuals allocate excess energy to storage and increase or enhance their body condition. By contrast, severe nutritional restriction may lead to malnutrition with subsequent reduced survival and reproductive failure (Simard et al. 2008). Changes in energy intake and expenditure can be affected by seasonal and annual variation in food availability (Bojarska & Selva, 2012), reproductive status (Rode et al. 2006), and climate change (Walther et al. 2002). Therefore, knowledge of the feeding ecology of an animal species and how its body condition changes as a result of environmental variation is critical to understand the ecology of the species and achieve effective management and conservation.

Some omnivore species consume a highly variable diet in response to spatial and temporal variation in food resources (Bojarska & Selva, 2012; Mowat & Heard, 2006; Vulla et al. 2009; Zalewska & Zalewski, 2019). However, brown bears (*Ursus arctos*), our model species, often rely on seasonally restricted, highly nutritious foods such as soft mast (e.g., *Vaccinium* sp. berries, a source of carbohydrate; Hertel et al. 2018) and hard mast (e.g., *Quercus* sp. acorns, a source of lipid; Naves et al. 2006), as well as seasonally available meats such as salmonid fish (a source of protein and lipid) that migrate upriver during the spawning season (Deacy et al. 2016). Dietary sources of lipids and carbohydrates play important roles in determining bear productivity, since bears use lipids and carbohydrates to adjust the protein contents of their diets to optimal levels for maximizing mass gain (Erlenbach et al. 2014; Robbins et al. 2007). The challenge is that mast production and upstream salmon abundance vary by year, and the resulting annual fluctuation in dietary content affects body condition, survival, and reproductive success, as well as movement and habitat selection of brown bears (Blanchard, 1987; Stenset et al. 2016; Welch et al. 1997; Zedrosser et al. 2006). For example, brown bears on the Kenai Peninsula depend on Pacific salmon (*Oncorhynchus* sp.) from June to October, and years when dietary salmon content during years is reduced correspond to years of decreased body fat in adult females (Hilderbrand et al. 1999). Bilberry (*Vaccinium myrtillus*) production from late July to late August drives the body mass gain and reproductive success of Swedish brown bears (Hertel et al. 2018), and the annual availability of crowberries (*Empetrum nigrum*) was negatively correlated with the use of salmon streams by brown bears in Katmai National Park (Rode et al. ,^2006^, ^2007^). Furthermore, in the Greater Yellowstone Ecosystem, whitebark pine (*Pinus albicaulis*) seeds account for the majority of the bear diet from about mid‐August through the end of September, and annual variation in nut production has been linked to changes in bear's use of land at different elevations (Blanchard & Knight, 1991). Key foods and their effects on the body condition and behavior differ depending on the habitat of bears, complicating the underlying causes of human–bear conflicts. In Yellowstone, annual fluctuations in pine nut abundance are associated with the number of incidents of bears damaging property and obtaining anthropogenic foods (Gunther et al. 2004), while fluctuating berry production does not alter bear behavior in Sweden (Hertel et al. 2019). The influence of dietary variability on body condition should be examined in various populations to determine the causes of human–bear conflicts and to understand how brown bear foraging strategies for temporarily limited food resources affect survival and reproduction.

**FIGURE 1:**
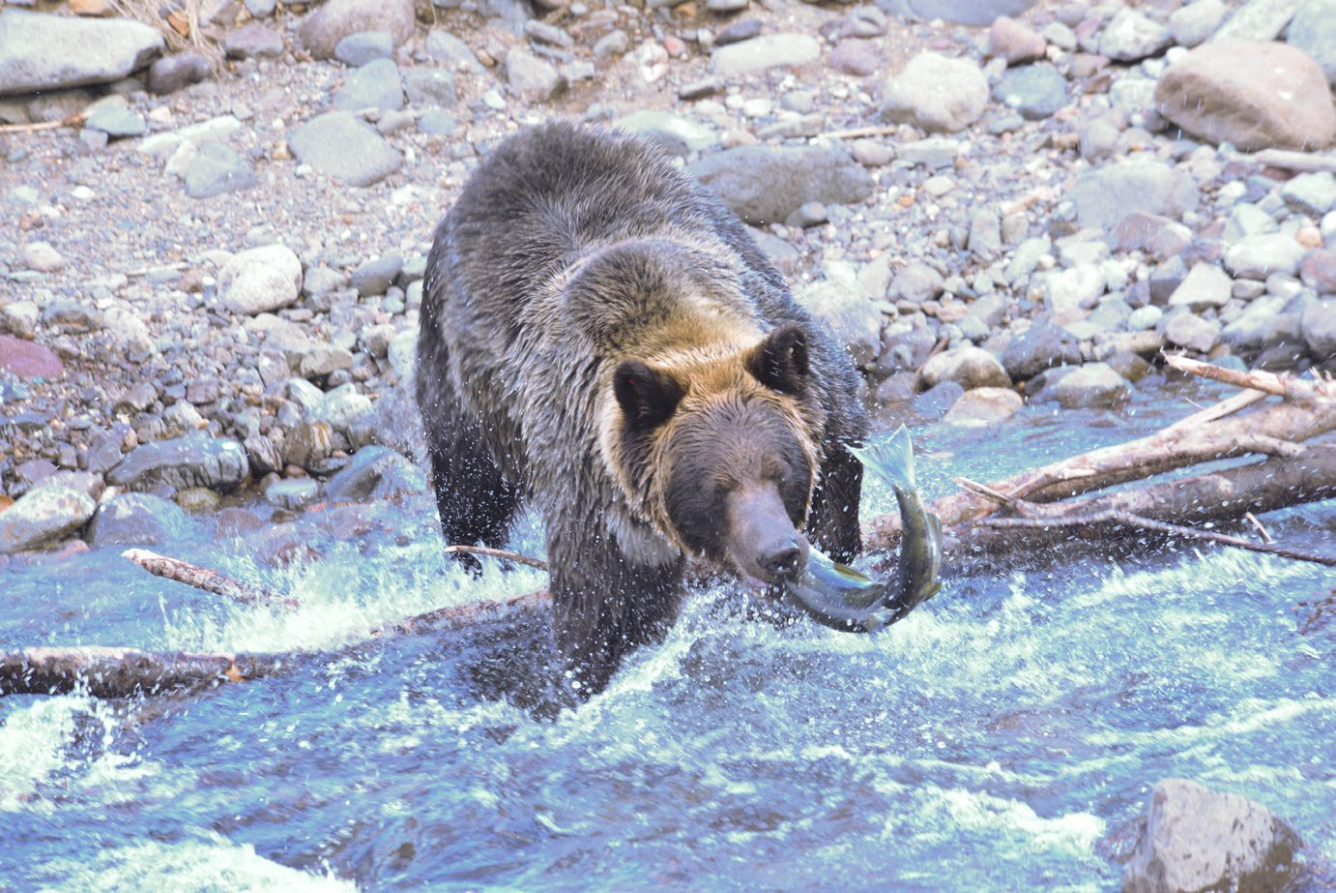
Brown bear catching salmon in the Rusha area of the Shiretoko Peninsula, Hokkaido, Japan (Photo: Masanao Nakanishi)

**FIGURE 2:**
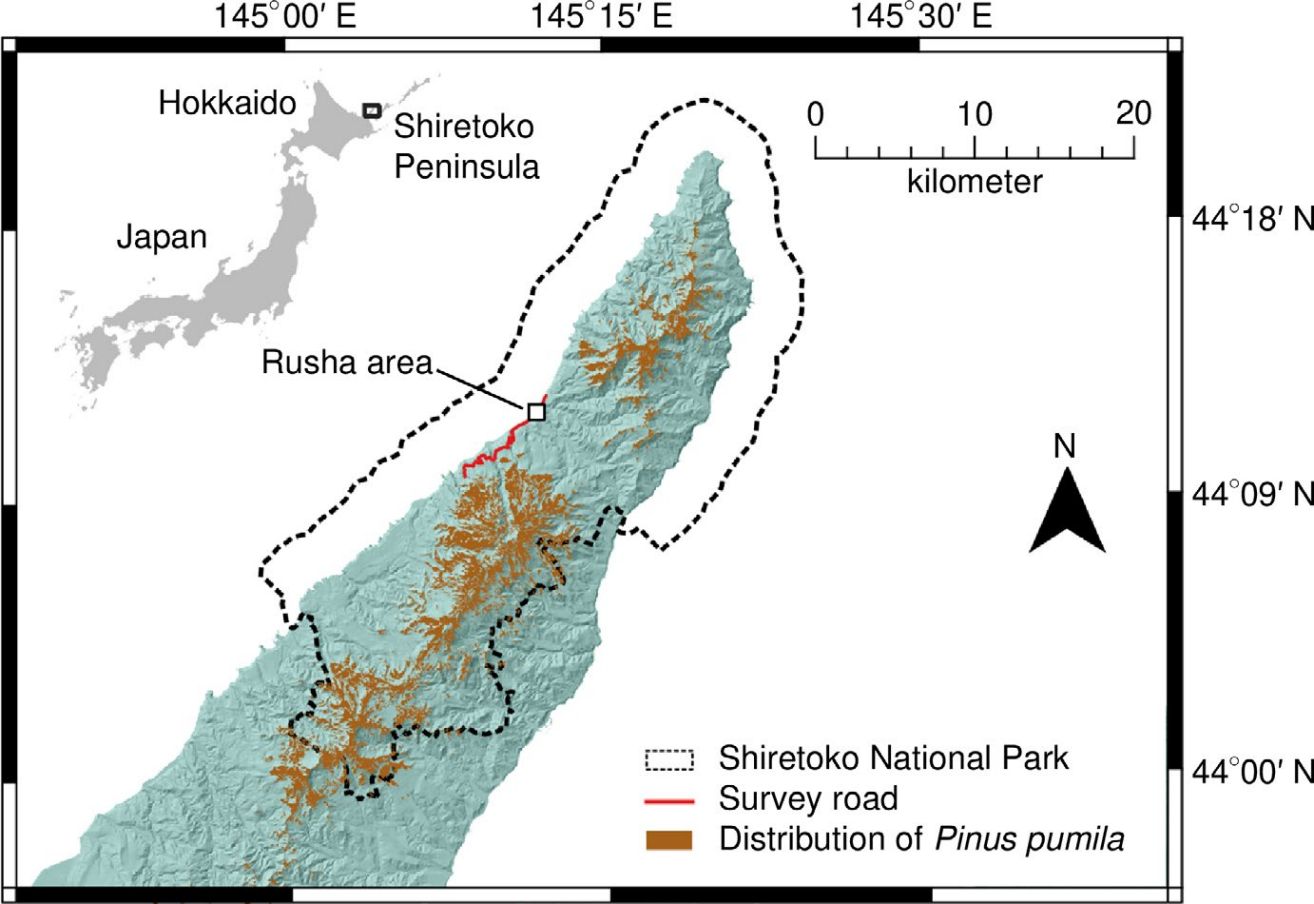
Map of the Shiretoko Peninsula, Hokkaido, Japan. This map was created using QGIS version 3.14.1 (QGIS Development Team, 2020. QGIS Geographic Information System. Open Source Geospatial Foundation Project. http://qgis.osgeo.org) and edited by the author. The topographic features are based on the National Land Numerical Information published by the Ministry of Land, Infrastructure, Transport, and Tourism of Japan (available from http://nlftp.mlit.go.jp/ksj/index.html, accessed 25‐Sep‐2020). The vegetation is modified from GIS data of 1:25,000 scale vegetation map created by Biodiversity Center of Japan, Ministry of the Environment (available from http://gis.biodic.go.jp/webgis/sc‐023.html, accessed 25‐Sep‐2020)

In Japan, brown bears exclusively inhabit Hokkaido, the northernmost island of the country (Figures 1 and 2). The Shiretoko Peninsula, located in eastern Hokkaido, has one of the highest densities of brown bear populations worldwide (Hokkaido Government, 2017). Although the Shiretoko Peninsula contains high‐quality brown bear habitat, human–bear conflicts, including agricultural crop depredation and intrusion into human residential areas, have become a serious problem. Over the past decade, an average of 34 bears have been killed each year, mainly for management purposes (Kohira et al. 2009; Shimozuru, Shirane, Yamanaka, et al., 2020). In 2012 and 2015, the number of bears killed for nuisance control was 65–67, nearly twice the usual number, and peaked in August (our unpublished data). During the same summer, several thin bears and starved cubs were observed, indicating that poor nutrition due to a lack of summer foods might cause bears to intrude into residential areas in search of food. In addition, Shimozuru et al. (2017) revealed that most cub disappearances on this peninsula occur in July and August (outside of the breeding season), which suggests that the main cause of cub mortality is deterioration of body condition in summer, rather than infanticide by adult males. Although Shiretoko brown bears have access to high‐energy foods such as Japanese stone pine (*Pinus pumila*) nuts in the subalpine zone and pink salmon (*Oncorhynchus gorbuscha*) spawning in the estuaries in summer (Ohdachi & Aoi, 1987), it remains unknown how much these food resources from completely different environments contribute to energy intake each month. It is also unclear whether food habits vary by year and how such variation affects the body condition of bears. For proper conservation and management of brown bear populations, it is important to determine which food resources determine the body condition of bears and whether food shortages affect the reproductive success of adult females.

The purpose of this study was to clarify seasonal and annual fluctuation in the body condition of adult female brown bears in relation to food habits and reproductive status. We conducted a 7 year longitudinal study that included scat sampling and direct observation of bears in a special wildlife protection area on the Shiretoko Peninsula. Male‐biased dispersal and female philopatry have been previously reported for brown bears on the Shiretoko Peninsula (Shirane et al.^2018^, ^2019^); thus, we investigated the contents of scat collected in the Rusha area to reflect the typical diet of female brown bears inhabiting the area. Using photographic evaluation to assess the body condition of free‐ranging brown bears (Shirane et al. 2020), we noninvasively monitored the body condition of identifiable bears throughout the study period. This study focused on mechanisms driving the timing of the inflection point at which the body condition turns from deterioration to recovery. Previous studies of bears have shown cyclical annual patterns in body condition: declining through spring to summer, beginning to increase concomitantly with the onset of high‐energy food consumption, and then peaking before hibernation (Hilderbrand et al. 2000; McLellan, 2011; Schwartz et al. 2014). In our study area, although data were available for only one individual, a seasonal fluctuation in body condition was reported from late June to early October, with the lowest condition in late August and the highest in early October (Shirane et al. 2020). Because annual fluctuations in the high‐energy food content of the diet can affect body condition (Hertel et al. 2018; Hilderbrand, Jenkins, et al., 1999), we predicted that (a) the timing of the body condition inflection point would be early, and the body condition would recover rapidly after the inflection point in years when the consumption of high‐energy foods in August and September was high. Female bears with dependent offspring have increased energy expenditure due to their investment in cub rearing (Wright et al. 1999) and have lower energy intake due to the low mobility of their young and consequent fewer foraging opportunities (Martin et al. 2013; Steyaert et al. 2014). Therefore, we predicted that (b) the timing of the inflection point would be earlier, and recovery would be more rapid in solitary females than in females with offspring.

## 2 MATERIALS AND METHODS

### 2.1 Ethical approval

Field experiments were approved by the Hokkaido Regional Environment Office and Kushiro Nature Conservation Office (Permit Number: 1606091 and 1705182). All procedures were conducted in accordance with the Guidelines for Animal Care and Use of Hokkaido University and were approved by the Animal Care and Use Committee of the Graduate School of Veterinary Medicine, Hokkaido University (Permit Numbers: 1106, 1151, 1152, 15009, 17005, and 18‐0083).

### 2.2 Study area

Shiretoko National Park extends from the center to the tip of the Shiretoko Peninsula (approximately 1,760 km^2^; Figure 2), eastern Hokkaido, Japan. In addition, it has been on the UNESCO World Natural Heritage List since 2005, being valued for its unique ecosystem formed by the interrelationship of its marine and terrestrial environments. The mountains in Shiretoko reach 1,500–1,600 m in height within 10 km of the coastline and generate a large number of steep slopes and streams. We performed field research in the Rusha area (44°11′–44°12′ N, 145°10′–145°12′ E, approximately 11.5 km^2^), which is located near the tip of the peninsula (Figure 2). This area is a narrow estuarine coast stretching south to north for approximately 3 km and has been designated as a special wildlife protection area where public access is prohibited without permission. No humans inhabit the area except for one fishing settlement, and the fishermen have not excluded bears from their settlements for the past few decades. For this reason, the bears have become habituated to and thus ignore humans, which allows us to observe bears directly at close range (Shimozuru, Shirane, Yamanaka, et al., 2020). In a previous study investigating bear reproductive parameters in the Rusha area, an average of 40 individuals were observed annually, including 15 adult females and three adult males (Shimozuru et al. 2017). Three streams where pink salmon and chum salmon (*Oncorhynchus keta*) spawn naturally flow into the sea within the Rusha area (Nakamura & Komiyama, 2010). The montane vegetation up to 500 m is characterized by mixed forests of coniferous and deciduous forests, for example, *Abies sachalinensis*, *Picea jezoensis*, *Picea glehnii*, *Quercus crispula*, *Acer pictum*, and *Alnus hirsuta*. Subalpine regions are mainly covered by *Betula ermanii*, and the area above the forest limit (approximately 570–1,100 m in height) is extensively covered by Japanese stone pine shrubs (Figure 2). In addition, local stone pine communities are also found in several locations along ridges in the montane zone (approximately 300–400 m in height). The Japanese stone pine is a mast species that produces good and poor seed production alternately on a 3‐ to 4‐year cycle (Nakashinden, 1995), but data on yearly fluctuations in pine nut crops on the Shiretoko Peninsula are limited.

### 2.3 Field methods

Periodic surveys (≥1 day/2 weeks) were conducted in the Rusha area during 2012–2018. Field teams patrolled survey roads in the area (approximately 3 km; Figure 2) by car and tracked bears when they appeared from the mountainside to observable places such as on the road or on the coast. We kept a distance of about 20–100 m from them to avoid interruptions or effects on their natural behavior. Individual bears were identified by field staff according to their appearance (e.g., size, color, facial characteristics, chest markings, and ear tags) as described in Shimozuru et al. (2017). This study focused on 12 adult female bears (≥5 years old; bear ID: BE, DC, DR, GI, HC, KB, KR, LI, RI, WD, WK, and WM) that could be easily identified and were frequently observed in the Rusha area throughout the surveillance period. When encountering these target bears, we took close‐up photographs from the lateral side using a digital single‐lens reflex camera. In addition, we defined three reproductive statuses of females: females accompanied by cubs, females accompanied by yearlings, and solitary females. When a cub disappeared from its mother, the cub was considered dead, as in other studies (Miller et al. 2003; Shimozuru et al. 2017; Swenson et al. 2001).

We collected fresh bear scats when encountered in the Rusha area, mainly in low‐altitude grasslands and coasts, and along survey roads leading to the area (approximately 9.5 km; Figure 2) from June to November in each year during 2012–2018. We recorded the collection date, location, and percent volume of each food item estimated visually (vFV) for each scat. We estimated the time from defecation to scat collection (≤2 weeks) based on freshness in relation to recent weather conditions (rain, sunshine, etc.) in order to classify the scats into 1 of 5 months from June to October. The field team in each survey included at least one of four core members who had extensive experience identifying the content of bear scat. In addition, scats encountered during 2013–2018 were collected individually in plastic bags and stored at –30°C for later analysis.

### 2.4 Laboratory analysis of diet

We analyzed scat samples, except those in 2012 that were not collected, using the point‐frame method (Sato et al. 2000). Each scat sample was filtered through a sieve (1.0 mm mesh) in running water. Because this method assumes a random distribution of each food item in the sampling population, materials remaining on the sieve were thoroughly mixed, and 30–90 g of them was evenly spread on a high‐sided, enameled laboratory tray. The bottom of the tray consisted of a 1 cm grid, and the points of intersection were considered point frames. We identified each food item lying on points of intersection on the tray and counted the number of intersections on which each food item lays. After completing the count of all food items contained in one scat sample, we calculated the occupancy of food item*_i_* as follows: Occupancy*_i_* [%] = 100 × (the number of intersections covered with food item*_i_*)/(the total number of intersections covered with all food items contained in the sample). We counted ≥200 points for each sample and used the occupancy as the volumetric proportion in subsequent analyses. For scat samples containing salmon, however, we could not count ≥200 points for most samples, because salmon is highly digestible and most fecal content was eliminated by washing. Thus, we made the following exception only for scats containing salmon: Even if the count number was <200 points, the calculated occupancy was included in the following analysis when the wet weight of the scat sample was ≥50 g. Nonfood items were those deemed to have been ingested incidentally by bears (e.g., anthill materials, twigs, wood fragments, needles from coniferous trees, and debris). Nonfood items also included bear hairs, with mean volumetric proportions per scat ≤0.5% (Ciucci et al. 2014), which were presumably ingested during grooming. To eliminate interobserver bias, we used two trained observers in the point‐frame analysis.

### 2.5 Quantification of diet

We first determined the percent frequency of occurrence and the percent fecal volume for each food item semimonthly and monthly. Because not all food items are digested to the same extent, food items that are more difficult to digest might be overestimated, and easily digestible food items might be underestimated (Hewitt & Robbins, 1996). To prevent such bias, we also estimated their contribution to the diet in terms of ingested dry mass and energy content. We used the corresponding correction factors (CF_D_, see Appendix A for details; Hewitt & Robbins, 1996; Dahle et al. 1998; Persson et al. 2001; Bojarska & Selva, 2013; Stenset et al. 2016) to calculate estimated dietary content (EDC). To calculate estimated digestible energy content (EDEC), we used another group of correction factors (CF_E_, see Appendix A for details; Mealey, 1980; Pritchard & Robbins, 1990; Dahle et al. 1998; Persson et al. 2001; Ciucci et al. 2014; Stenset et al. 2016) We did not calculate EDC or EDEC for nonfood items. We summarized EDC and EDEC values by nine categories: plants (leaves of herbaceous plants and broadleaf trees), pine nuts, drupes, berries, acorns and other nuts, insects, mammals, salmon, and "other."

In this study, we collected scat samples during 2013–2018 and calculated EDC using the results of point‐frame analysis; however, we did not bring scat samples back to the laboratory in 2012, so scat content data were available only as vFV for 2012. To report more accurate results, we presented seasonal changes in diet based on EDC values from samples collected during 2013–2018. In addition, to include the 2012 data in the comparison of annual changes in diet, we used a linear regression with no intercept to verify the validity of the visual estimates and to correct the vFV so that these data could be used as equivalent to the EDC. For more details on the analysis, please see Appendix B.

### 2.6 Estimation of body condition

Following Shirane et al. (2020), we assessed the body condition of adult female brown bears using morphometric measurements from photographs. Shirane et al. (2020) confirmed that this method accurately reflects true body condition (i.e., the body condition index obtained from the regression of body mass against body length) and has high measurement precision between photographs. However, this photograph‐based method is limited by its requirement for photographs with sufficient quality for morphometric measurements and bear posture that does not affect the evaluation. To overcome this, we first graded lateral photographs of each individual bear based on several attributes for photograph condition and bear posture (see Appendix B for details; Shirane et al. 2020). Photographs were scored 1 (good quality), 2 (medium quality), or 3 (poor quality) for each attribute, and those with a score of 3 for any attribute were removed from subsequent analyses. Then, we used ImageJ version 1.52a (Schneider et al. 2012) to extract morphometric measurements from lateral photographs of bears following the protocols described in Shirane et al. (2020). Specifically, first we adjusted the angle of all the photographs according to the ground surface; then, we measured the torso height (TH) and the horizontal straight‐line torso length (HTL) in pixels: TH was the distance perpendicular to the ground from the lowest point of the abdomen to the highest point of the waist, and HTL was the straight‐line distance from the base of the tail to the highest part of the shoulder parallel to the ground. TH and HTL were measured three times per photograph, and the ratio of TH to HTL (TH:HTL) was calculated from the respective average values. For each of eight sessions (June, July, early August, late August, early September, late September, October, and November), we calculated TH:HTL using at least two photographs per individual, and the median of these was used as an indicator of body condition. We included bear‐years with TH:HTL data for at least two out of eight sessions per year in the analyses. Bear‐years for which TH:HTL data were available for only one session in a particular year were excluded from the analyses. In addition, if reproductive status affects the body condition of female bears, their body condition may differ before and after the loss of offspring. To eliminate this effect, when we observed females that had lost their dependent young, either by death or by family break‐up, any data for subsequent sessions of these females were excluded from the following analyses.

### 2.7 Statistical analyses

We did not identify which individual bear scat came from or whether the scats were from females. Given the known philopatric nature of females on the Shiretoko Peninsula (Shirane et al. 2018) and the fact that adult males were rarely observed in the Rusha area (Shimozuru et al. 2017), we assumed for our analyses that the average content of the scats collected during each period was typical of adult females in the Rusha area. To test our first prediction that in years with high consumption of high‐energy foods in August and September, the timing of the inflection point in body condition would be earlier, and body condition would recover more rapidly after the inflection point, we distinguished years with high or low consumption of high‐energy foods. Based on the results of seasonal changes in diet (detailed below), we focused on pine nuts as the only high‐energy food available in August, and on salmon in September. We classified years as having either high or low consumption of pine nuts and salmon depending on whether the EDC values in August and September during 2012–2018 exceeded the mean EDC.

We used generalized additive mixed models (GAMMs; smoothing analyses) (Zuur et al. 2009) to identify nonlinear effects of session on body condition and the relationships among body condition, dietary content, and reproductive status. In each GAMM, the response variable (TH:HTL) was run with a gamma family distribution and log link. The year and bear ID were included in the intercept as a crossed random effect because the same bears were sampled across several years. Because there are previously reported effects of month of year on bear body condition in our study area (Shirane et al. 2020), a null model without session would not make biological sense. Hence, we included session (starting from June: 1, 2, 3, 3.5, 4, 4.5, 5, and 6) in all models. We used thin‐plate regression splines to fit session, in which the beginning and end points of a cycle were not constrained by each other (Zuur et al. 2009). Possible predictor variables included in models were session (smooth term), reproductive status (categorical: solitary female or female with dependent young), diet 1 (categorical: high consumption or not of both pine nuts and salmon), diet 2 (categorical: low consumption or not of both pine nuts and salmon), and the interaction between reproductive status and diet. Because Hilderbrand et al. (2000) reported that body weight and percent body fat were similar in brown bear mothers with cubs and yearlings, and both were lower than those in solitary females, we classified reproductive status only as solitary or with dependent young as an explanatory variable. Model selection involved comparing Akaike's information criterion corrected for small sample size (AIC_c_) values between a set of ecologically relevant candidate models defined a priori (Burnham & Anderson, 2002). All statistical analyses were performed in R 4.0.2. (R Core Team, 2020) with the lme4 (Bates et al. 2015), emmeans (Lenth, 2020), and mgcv (Wood, 2011) packages. We validated the statistical models by plotting model residuals versus the fitted values (Zuur et al. 2009).

## 3 RESULTS

### 3.1 Scat collection and analysis

**FIGURE 3:**
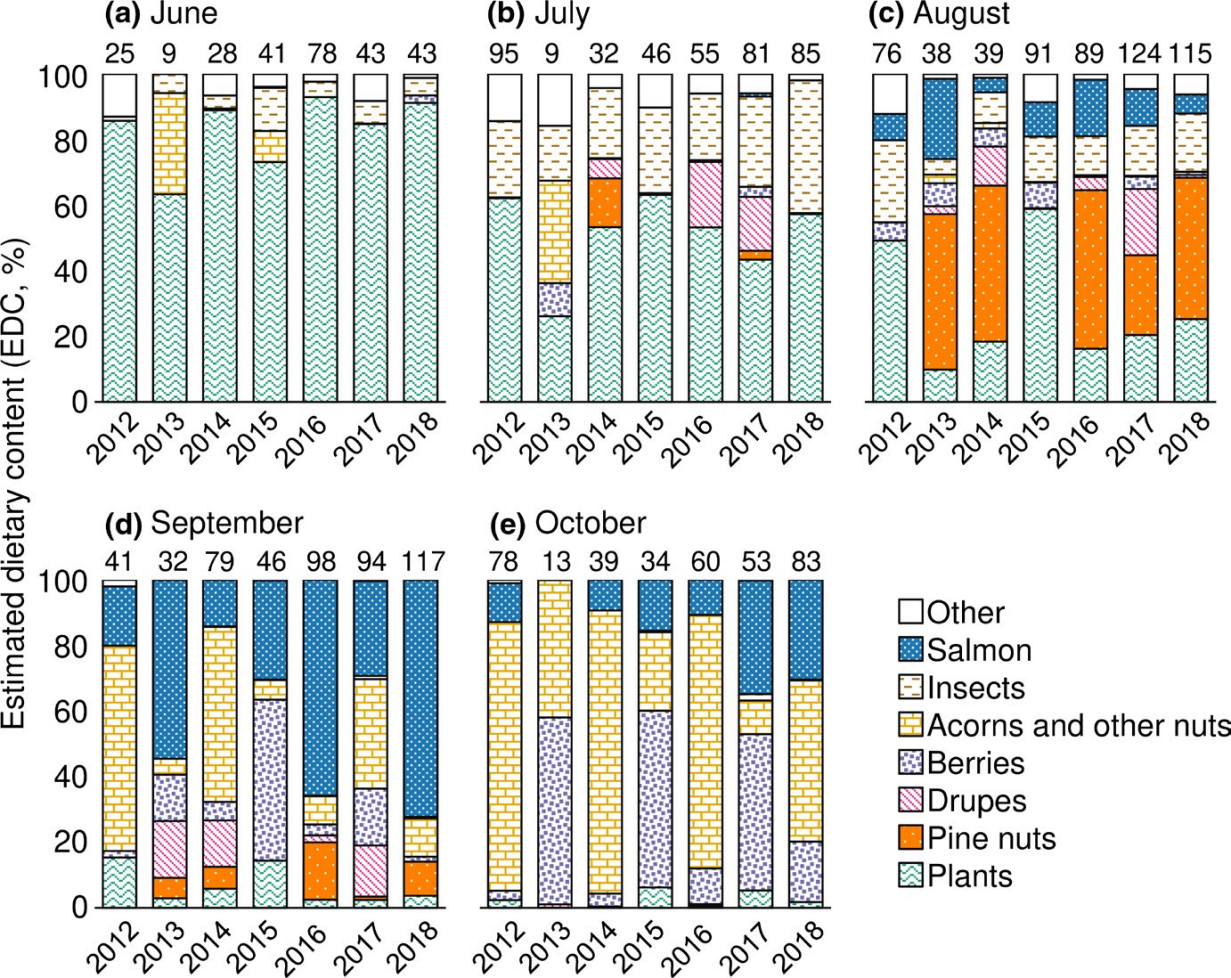
Annual variation in the estimated dietary content (EDC) of 2,079 brown bear scat samples collected in the Rusha area of the Shiretoko Peninsula, Hokkaido, Japan, during 2012–2018. The numbers above the figure represent the number of scat samples

We collected a total of 2,079 scats (267, 403, 552, 507, and 350 for each month from June to October, respectively), including 315 analyzed visually in 2012 and 1,764 analyzed using the point‐frame estimation during 2013–2018. We presented seasonal variation in diet (Table 1) based on EDC values from samples collected during 2013–2018, and annual variation in diet (Figure 3) based on EDC during 2013–2018 and vFV in 2012 (corrected based on linear regression between EDC and vFV during 2013–2018; Appendix C).

**TABLE 1:** The mean seasonal diet of brown bears in the Rusha area based on analyses of 1,764 fecal samples collected during 2013–2018

Food item^a^	June (*n* = 242)		July (*n* = 308)		August (*n* = 476)		September (*n* = 466)		October (*n* = 272)	
	EDC	EDEC	EDC	EDEC	EDC	EDEC	EDC	EDEC	EDC	EDEC
**Plants**	76.4	60.8	47.7	33.1	24.7	14.8	5.3	2.6	2.4	1.1
Herbaceous plants	70.4	56.0	46.4	32.1	24.2	14.6	5.2	2.6	2.3	1.1
Woody plants	5.9	4.8	1.3	0.9	0.5	0.3	*tr*	*tr*	0.1	*tr*
Seaweed	*tr*	*tr*	0.1	*tr*	*tr*	*tr*	—	—	—	—
**Pine nuts**										
*Pinus pumila*	—	—	2.8	4.2	34.9	39.8	7.0	7.2	—	—
**Drupes**	*tr*	*tr*	6.9	5.2	6.6	5.2	8.2	6.4	0.3	0.2
*Prunus sargentii*	—	—	5.9	4.3	0.4	0.2	—	—	—	—
*Prunus ssiori*	—	—	1.0	0.8	4.4	3.0	4.5	2.7	0.2	0.1
**Berries**	0.3	0.6	2.4	3.2	4.2	5.2	15.2	16.3	31.8	32.4
*Vaccinium* spp.	—	—	0.1	0.1	*tr*	*tr*	—	—	—	—
*Morus australis*	—	—	0.5	0.7	0.1	0.1	—	—	—	—
*Vitis coignetiae*	—	—	*tr*	*tr*	2.1	2.6	7.8	8.4	5.7	5.8
*Sorbus commixta*	—	—	—	—	1.0	1.1	3.3	3.5	4.4	4.5
*Actinidia* spp.	—	—	—	—	0.5	0.7	2.7	2.9	17.1	17.4
*Aralia* spp.	—	—	—	—	*tr*	*tr*	0.9	1.0	—	—
*Phellodendron amurense*	—	—	—	—	—	*tr*	0.3	0.3	3.3	3.4
**Acorns and other nuts**	6.7	10.3	5.2	6.3	0.9	1.0	19.6	20.8	47.4	47.8
*Quercus crispula*	6.6	10.1	5.2	6.3	0.8	0.9	14.1	14.9	45.1	45.5
*Juglans mandshurica*	0.1	0.1	—	—	0.1	0.1	5.4	5.8	2.2	2.2
**Insects**	6.3	10.5	24.6	36.4	12.1	14.8	0.3	0.4	0.4	0.5
Formicidae	5.3	9.0	23.0	34.0	11.3	13.9	0.1	0.1	—	—
Vespidae	0.2	0.3	0.1	0.1	0.4	0.5	0.2	0.2	0.1	0.1
Diptera	0.7	1.1	0.8	1.3	0.1	0.1	*tr*	*tr*	—	—
**Mammals**	6.9	12.0	3.0	4.5	0.5	0.6	0.2	0.2	1.0	1.1
*Cervus nippon yesoensis*	6.1	10.7	2.0	2.9	0.3	0.4	0.1	0.1	0.8	0.8
*Ursus arctos* ^b^	*tr*	0.1	0.5	0.8	0.2	0.1	—	*tr*	*tr*	*tr*
**Salmon**										
*Oncorhynchus* spp.	—	—	0.1	0.1	12.2	14.4	44.2	46.1	16.7	17.0
Other	3.4	5.8	7.2	7.0	4.0	4.1	0.1	0.1	tr	tr
Fungi	0.1	0.1	3.3	1.3	0.8	tr	—	—	—	—
Birds	0.9	1.5	0.2	0.3	0.6	0.8	—	tr	—	—
Shellfish	0.9	1.5	1.6	2.3	1.4	2.0	tr	tr	tr	tr
Amphipoda	1.6	2.7	2.0	2.9	1.0	1.3	0.1	0.1	—	—

Data are estimated dietary content (EDC) and estimated dietary energy content (EDEC). Contributions < 0.05% are indicated by “*tr*” (trace) for clarity.

^a^ Macro categories include unidentified items at higher taxonomic levels.

^b^ Excluding scats with volumetric proportions < 0.5% to minimize inclusion of hairs from grooming.

### 3.2 Seasonal and annual variation in diet

We observed a trend of monthly changes in bear diet (Table 1 and Figure 3). Plants (leaves of herbaceous plants and broadleaf trees) consistently dominated the diet in June and July, with peak consumption in June. Insects (primarily ants) contributed an average of 36.4% EDEC in July, when their monthly consumption was highest. Mammals (primarily sika deer, *Cervus nippon yesoensis*) were also used, with peak consumption in June, although their contribution to EDEC was only 12.0%. Consumption of overwintering acorns in June and July was generally low. In August, although bears continued to consume plants and insects, nuts of Japanese stone pine dominated the diet, providing an average of 39.8% EDEC. In addition, salmon began to be consumed by bears in August, accounting for the fourth largest contribution to EDEC (14.4%). Salmon consumption was highest in September and contributed an average of 46.1% EDEC. Acorns and berries were also consumed from September, with peak consumption in October. The “Acorns and other nuts” category comprised almost exclusively *Quercus crispila* acorns, whereas the berries category included various species such as wild vine (*Vitis coignetiae*) and hardy kiwi (*Actinidia* spp.). On average, the major consumption periods for pine nuts and salmon were August and September, respectively.

We observed annual differences in bear diet in August and September (Figure 3). Based on the EDC of pine nuts in August, we identified 2013 (47.4%), 2014 (47.5%), 2016 (47.3%), and 2018 (42.9%) as years with above‐average consumption (34.9%), and thus, we considered them high‐consumption years. Similarly, we identified 2013 (54.4%), 2016 (65.4%), and 2018 (72.2%) as years with large consumption of salmon; all of these years also involved high consumption of pine nuts.

**TABLE 2:** Abbreviated name and description of factors included in generalized additive mixed models to the body condition (TH:HTL) of adult female brown bears in the Rusha area of the Shiretoko Peninsula, Hokkaido, Japan

Abbreviated factor name	Type	Description
Session	Smooth term	Numeric factor where 1, 2, 3, 3.5, 4, 4.5, 5, and 6 represent June, July, early August, late August, early September, late September, October, and November, respectively
RST	Fixed variable	Categorical factor where "1" indicates a female was solitary and "2" indicates she was accompanied by cubs or yearlings
Diet 1	Fixed variable	Categorical factor where "1" indicates years with high consumption of both pine nuts and salmon (2013, 2016, and 2018) and "2" indicates other years (2012, 2014, 2015, and 2017)
Diet 2	Fixed variable	Categorical factor where "1" indicates years with low consumption of both pine nuts and salmon (2012, 2015, and 2017) and "2" indicates other years (2013, 2014, 2016, and 2018)
Year	Random factor	Categorical year from 2012 to 2018
ID	Random factor	Categorical factor where "1"–"12" represent 12 adult female bears each

**TABLE 3:** Akaike's information criterion corrected for small sample size (AIC_c_), ΔAIC_c_, and within‐stage Akaike weights (*w_i_*) for model selection for factors influencing the body condition (TH:HTL) of adult female brown bears in the Rusha area of the Shiretoko Peninsula, Hokkaido, Japan

Models	AIC_c_	ΔAIC_c_	*w_i_*
S(Session × Diet 1) + *F*(RST) × *F*(Diet 1) + R(Year) + R(ID)	−1,247.1	0.0	0.4
S(Session × RST) + *F*(RST) × *F*(Diet 2) + R(Year) + R(ID)	−1,246.3	0.7	0.3
S(Session × RST) + *F*(RST) × *F*(Diet 1) + R(Year) + R(ID)	−1,244.1	3.0	0.1
S(Session × RST) + *F*(RST) + R(Year) + R(ID)	−1,244.0	3.1	0.1
S(Session) + *F*(RST) × *F*(Diet 2) + R(Year) + R(ID)	−1,242.3	4.8	0.0
S(Session) + *F*(RST) × *F*(Diet 1) + R(Year) + R(ID)	−1,241.8	5.2	0.0
S(Session × Diet 2) + *F*(RST) × *F*(Diet 2) + R(Year) + R(ID)	−1,241.3	5.8	0.0
S(Session) + *F*(RST) + R(Year) + R(ID)	−1,241.3	5.8	0.0
S(Session × Diet 1) + *F*(Diet 1) + R(Year) + R(ID)	−1,225.9	21.2	0.0
S(Session) + *F*(Diet 1) + R(Year) + R(ID)	−1,221.7	25.4	0.0
S(Session) + R(Year) + R(ID)	−1,220.0	27.1	0.0
S(Session) + *F*(Diet 2) + R(Year) + R(ID)	−1,219.7	27.4	0.0
S(Session × Diet 2) + *F*(Diet 2) + R(Year) + R(ID)	−1,218.1	29.0	0.0

S: smooth term.

F: fixed variable.

R: random intercept.

×: interaction between two factors.

### 3.3 Factors affecting body condition

We analyzed a total of 1,226 photographs of 12 adult females during 2012–2018. On average, 3.6 photographs of each individual were used per session. When data for all years during 2012–2018 were pooled, at least nine of the 12 females were evaluated for body condition in each session. See Appendix F for examples of morphometric measurements and body condition evaluation using photographs.

**FIGURE 4:**
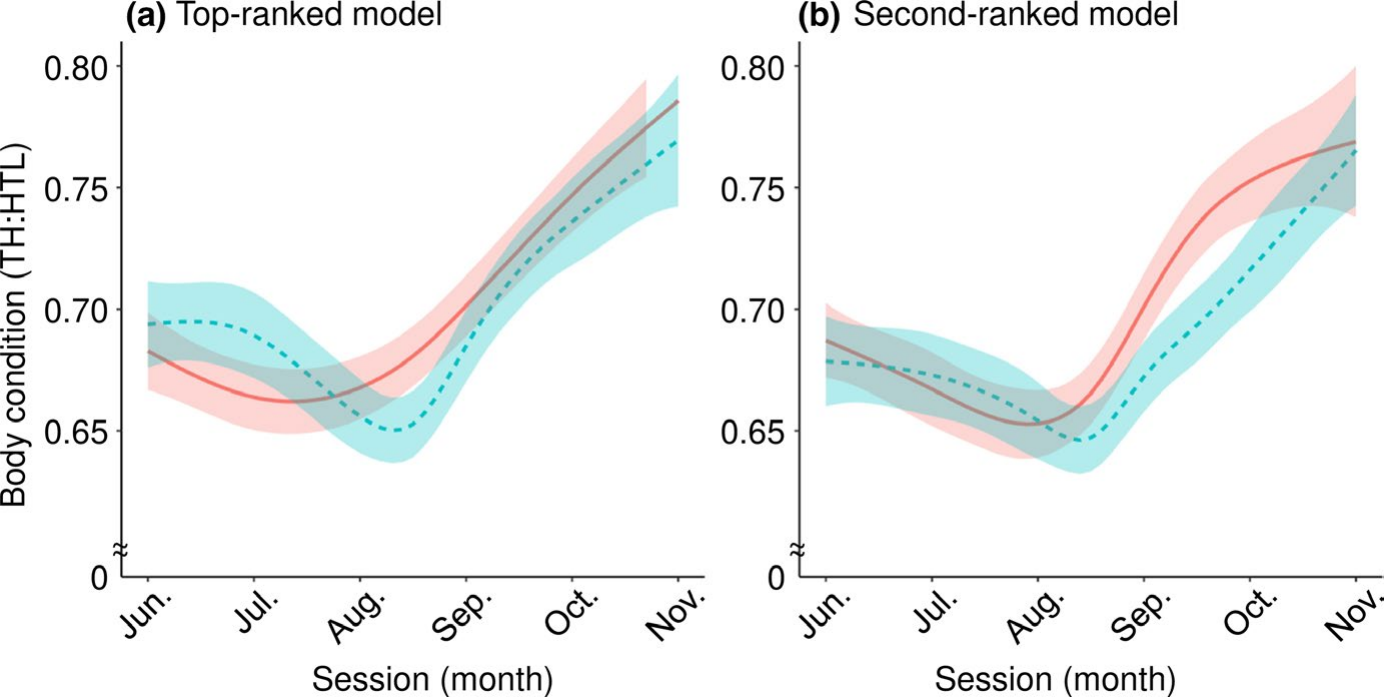
Seasonal changes in body condition predicted by generalized additive mixed models for adult female brown bears in the Rusha area of the Shiretoko Peninsula, Hokkaido, Japan. (a) The figure of the top‐ranked model. The red solid line indicates high consumption of both pine nuts and salmon, whereas the blue dotted line indicates low salmon consumption. (b) The figure of the second‐ranked model. The red solid line indicates solitary females, whereas the blue dotted line indicates females with dependent young. Lines represent mean estimates, and shaded regions represent 95% confidence intervals

Only the top two GAMMs had ΔAICc values < 2 (Tables 2 and 3). The top‐ranked model included reproductive status, diet 1 (high consumption or not of both pine nuts and salmon), and the interactions between session and diet 1 and reproductive status and diet 1. TH:HTL exhibited a general decline from June to August, followed by an increase from September to November (Figure 4a). In years with high consumption of both pine nuts and salmon, bears experienced smaller seasonal fluctuations in TH:HTL (*edf* = 2.90; Table 4): TH:HTL declined very little, reaching the lowest point in mid‐July and starting to recover in August. However, TH:HTL continued to decline until late August when salmon consumption was low (*edf* = 5.10; Table 4). The second‐ranked model included reproductive status, diet 2 (low consumption or not of both pine nuts and salmon), and interactions between session and reproductive status and reproductive status and diet 2. The overall seasonal pattern was similar to that of the top‐ranked model (Figure 4b). The timing of the inflection point at which body condition changed from deteriorating to recovering was not as pronounced as in the top‐ranked model, but tended to be earlier for solitary females (early August) than for females with offspring (late August). Differences due to reproductive status were more pronounced from late August to September, with solitary females recovering body condition more rapidly than females with dependent young. See Appendices F and G for standard diagnostic plots verifying the fitting procedure of the GAMMs.

**TABLE 4:** Summary of parameter estimates for the top two generalized additive mixed model fit to the body condition (TH:HTL) of adult female brown bears in the Rusha area. A significant *F* indicates nonlinearity. *edf* = estimated degrees of freedom

Variable	Parameter estimates				Significance of smooth term		
	*β*	*SE*	*t*	*p*	*edf*	*F*	*p*
Top‐ranked = TH:HTL ~ S(Session × Diet 1) + *F*(RST) × *F*(Diet 1) + R(Year) + R(ID)							
Intercept	−0.386	0.008	−49.5	<0.001	—	—	—
RST (with young)	−0.025	0.010	−2.6	0.011	—	—	—
Diet 1 (low consumption)	−0.010	0.008	−1.2	0.224	—	—	—
RST: diet 1	−0.009	0.013	−0.7	0.504	—	—	—
Session × High consumption	—	—	—	—	2.90	50.66	<0.001
Session × Low consumption	—	—	—	—	5.10	33.61	<0.001
Second‐ranked = TH:HTL ~ S(Session × RST) + *F*(RST) × *F*(Diet 2) + R(Year) + R(ID)							
Intercept	−0.389	0.008	−48.9	<0.001	—	—	—
RST (with young)	−0.023	0.010	−2.4	0.017	—	—	—
Diet 2 (low consumption)	−0.009	0.008	−1.1	0.265	—	—	—
RST: diet 2	−0.010	0.013	−0.8	0.431	—	—	—
Session × Solitary	—	—	—	—	4.44	37.10	<0.001
Session × With young	—	—	—	—	4.92	31.11	<0.001

S: smooth term.

F: fixed variable.

R: random intercept.

×: interaction between two factors.

## 4 DISCUSSION

This report is the first to quantify in detail the food habits of brown bears throughout the year on the Shiretoko Peninsula. Shiretoko bears mainly use herbaceous plants from spring to summer and berries and acorns in autumn, which is similar to patterns reported in brown bear populations around the world (Mattson et al. 1991; McLellan & Hovey, 1995; Stenset et al. 2016). In late summer, when the nutritional value of herbaceous plants decreases (Cicnjak et al. 1987) and berries are still immature, bears eat a variety of foods depending on population, including premature herbaceous plants in cooler areas (Munro et al. 2006; Rodríguez et al. 2007) and alternative foods such as anthropogenic foods (Piédallu et al. 2016; Sato et al. 2005). Our findings confirmed that pine nuts and salmon contributed a high percentage to the diet of brown bears on the Shiretoko Peninsula in August and September, respectively. This is not consistent with the food habits of bears in other Hokkaido populations that rely on herbaceous plants, berries, or crops in late summer (Aoi, 1985; Matsubayashi et al. 2014; Ohdachi & Aoi, 1987; Sato et al. 2004, 2005; Sato & Endo, 2006). Although consumption of pine nuts was also reported in a brown bear population on Mt. Daisetsu (with a summit reaching an altitude of 2,000 m; Ohdachi & Aoi, 1987), our study demonstrates that brown bears on the Shiretoko Peninsula are unique in that they consume both pine nuts and salmon. In addition, considering that altitudes of about 100 m are the upper limit of salmon run‐up in Hokkaido (Urabe et al. 2013), we have demonstrated that brown bears on the Shiretoko Peninsula depend on food in extremely different environments such as coasts and the subalpine zone in late summer. Scat samples collected in the Rusha area along the coastline contained a large amount of pine nuts, which suggests that brown bears frequently travel between coastal areas and subalpine zones in late summer and do not solely feed on pine nuts during random encounters.

The combination of scat analysis and photographic evaluation of body condition proved useful for noninvasive long‐term monitoring of free‐ranging brown bears. Although the 12 individuals assessed for body condition provide a relatively small sample size, this is one of few studies that have repeatedly monitored live large mammals using a noninvasive, photographic technique. Scat samples permit identification of the species of food item and quantification of the dietary content of bears. This made it possible to focus on specific food items, pine nuts and salmon, and compare their consumption by season and year. Although a limitation of scat analysis is that results cannot be linked to specific individuals without integration with direct observations or genetic analysis, we assumed that scat contents reflected the typical diets of philopatric adult females in the Rusha area. This assumption is reasonable because opportunities for the observation of adult males were rare compared with those for the observation of adult females in the Rusha area, and the female‐to‐male ratio of observed individuals in previous studies was 5:1 (Shimozuru et al. 2017). This imbalance is mainly due to the fact that adult male bears, which migrate from different regions, are not accustomed to the presence of humans, and thus, male bears are more wary of humans. In addition, we previously identified individuals via genetic analysis of 118 scat samples (12 of them containing pine nuts) collected in the Rusha area (Shimozuru, Shirane, Jimbo, et al., 2020), of which 94 samples were identified as originating from either adult females or their dependent young. Based on this information, we consider the content of scat collected in the Rusha area to reflect the typical food habits of the 12 female brown bears targeted in this study.

Our study provides insight into seasonal patterns in body condition in brown bears in relation to food condition dynamics. We found evidence that body condition continued to decline in late August and then increased to a peak before denning, which indicates that fluctuations were related to seasonal resource availability and increased investment in foraging. The period of time between bears emerging from their dens and the maturation of fruits has been called the “negative foraging period” (Noyce & Garshelis, 1998). Many wild bears lose fat and lean tissue during this time period (Blanchard, 1987; Eagle & Pelton, 1983; Hellgren et al. 1989) because bears can feed only on high‐protein but low‐energy herbaceous foods (Rode et al. 2001) and face the costs of maintenance, growth, and cub rearing (Hilderbrand et al. 2000). Similar to previous studies, our findings suggest that available spring to early summer food resources (i.e., plants and insects) provide insufficient energy for Shiretoko brown bears to maintain their body condition after den emergence. In September, when salmon consumption increased, body condition started to increase as predicted. Around the same time, brown bears were consuming a variety of high‐energy foods, such as soft and hard mast, and rapidly depositing fat. Although salmon consumption decreased and acorns dominated the diet in October, body condition continued to improve until November, which indicates that increased intake of a high‐energy but low‐protein diet contributes extra energy to fat deposition, as suggested by Felicetti et al. (2003).

The body condition of the bears began to recover later on the Shiretoko Peninsula (i.e., in September) compared with bear populations in British Columbia (in August; McLellan, 2011) and Yellowstone National Park (in July; Schwartz et al. 2014). Hokkaido brown bears emerge from their dens between March and May, which is similar to the timing of den emergence in British Columbia (in April–May; Ciarniello et al. 2005) and Yellowstone (in late march; Judd et al. 1986). Therefore, the period of poor body condition is longer in Hokkaido brown bears than in other populations. This difference may depend on when high‐energy foods become available in summer. British Columbia and Sweden, located at higher latitudes than Shiretoko, have different plant phenology in which brown bears can obtain an abundance of mature fruits from early summer (McLellan & Hovey, 1995; Stenset et al. 2016). Although Yellowstone and Shiretoko share a common plant phenology in that pine nuts are not available until late summer (Mattson et al. 1991), the different timings of salmon runs in these regions allow salmon to play different roles for brown bears. Cutthroat trout (*Oncorhynchus clarki*) running upstream and entering Yellowstone Lake in early summer may provide bears the opportunity to regain body condition after den emergence and help females with cubs meet the energetic demands of lactation (Mattson & Reinhart, 1995; Reinhart & Mattson, 1990), while pink salmon spawning in estuaries in late summer may act as an accelerator for the recovery of poor body condition on the Shiretoko Peninsula. In addition, brown bears are important predators of ungulate neonates in North America during spring, whereas the consumption of ungulates during the parturition season of sika deer (June; Suzuki et al. 1996) was limited in this study. Although the Shiretoko Peninsula harbors diverse and abundant food resources for brown bears, the availability of high‐quality foods occurs primarily during autumn; therefore, they likely experience a particularly harsh summer compared with brown bears in other locations.

Annual differences in dietary content created different seasonal patterns in body condition. Seasonal patterns in body condition differed between years when both pine nut and salmon consumption were high and years when they were not. In the years with high consumption of both food items, body condition began to recover earlier, resulting in a better summer body condition. Our results support our prediction that the high consumption of high‐energy foods in late summer would lead to an earlier inflection point, but do not support our prediction that recovery would be more rapid. Even in years with high pine nut consumption and low salmon consumption (2014), body condition exhibited the same seasonal pattern as in years when the consumption of both was low (2012, 2015, 2017). Because our data set obtained during 2012–2018 did not contain years when consumption of pine nuts was low and salmon consumption was high, we cannot determine whether the difference in body condition seasonality was caused by salmon alone or both salmon and pine nuts. However, considering that body condition began to recover in August in years with good food conditions, it is reasonable to assume that pine nuts in August also contribute to the recovery of body condition. Eating a large amount of both pine nuts and salmon enables rapid recovery, despite the aforementioned harsh summers of the Shiretoko Peninsula.

As we predicted, the timing of the inflection point tended to be earlier, and the recovery, more rapid, in solitary females than in females with offspring. We propose three potential reasons for this. First, pregnant bears must invest in their cubs to give birth and subsequently to lactate (i.e., high energy expenditure). Females with cubs of the year have a lower lean body mass than solitary females in the spring (Hilderbrand et al. 2000), with increased costs of protein catabolism due to lactation demands (Wright et al. 1999). Second, females with cubs of the year can move only limited distances in search of food resources (i.e., low energy intake). The movement rate of adult female brown bears in Sweden is slower when they are accompanied by cubs than when they are solitary (Martin et al. 2013; Steyaert et al. 2014). Although our results suggest that brown bears frequently travel between the subalpine region and the coastline for foraging, further research is needed to clarify whether such movement is even possible for females with cubs and whether habitat selectivity differs depending on reproductive status. Third, mother bears have limited opportunities to forage for salmon, which could explain the difference in September recovery patterns between solitary females and females with dependent young. Females with dependent young tend to avoid salmon spawning streams to reduce the risk of infanticide by adult males (Ben‐David et al. 2004) and to provide security for their young from socially dominant conspecifics (Egbert et al. 1976; Gende & Quinn, 2004).

Our results demonstrate that both dietary content and reproductive status are the primary determinants of seasonal and annual variation in the body condition of brown bears. The two top‐ranked models indicated that females with offspring exhibit particularly poor condition in late August in years with low consumption of pine nuts or salmon. This finding has important implications not only for seasonal and annual fluctuation in body condition but also for the long‐term survival of the Shiretoko brown bear population. In the Rusha area, the worst season for body condition (August) coincided with the period with the highest mortality rates for cubs of the year (Shimozuru et al. 2017). Our results are consistent with the claim that cub mortality is mainly due to poor nutrition in summer rather than infanticide by adult males in the Rusha area. Since this study was only able to obtain body condition data for 12 individuals, future studies will need to increase the sample size in order to determine the effects of body condition of mothers and offspring on markers of reproductive success such as litter size and cub mortality. For polar bears (*Ursus maritimus*), less sea ice due to global warming reduces the seal hunting opportunities, resulting in lighter body mass of female polar bears and fewer offspring (Amstrup et al. 1986; Derocher et al. 2011; Stirling & Derocher, 1993). Therefore, it is necessary to continue to monitor long‐term trends in the food environment and body condition of brown bears to better understand population dynamics on the Shiretoko Peninsula.

Our findings may help to clarify the causes of the human–bear conflicts. Previous studies of bears have shown that the incidence of human–bear conflict increases in response to reduced food availability rather than increased population size (Arimoto et al. 2011; Kozakai et al. 2011; Mattson et al. 1992; Su et al. 2018). The present study revealed that the two years with increased human–bear conflict on the Shiretoko Peninsula (2012 and 2015) was consistent with the low consumption of both pine nuts and salmon, suggesting that summer energy shortages may lead to bear intrusion into residential areas. However, the results showed that bears exhibited poor summer body condition not only during 2012 and 2015, but also in other years, indicating that malnutrition is not the sole factor causing bears to intrude into human settlements. Yamanaka (2011) suggested that the feeling of hunger that occurs regardless of a bear's nutritional status may lead to bear intrusion into residential areas. The low consumption of pine nuts and salmon in the present study may support this theory. On the other hand, Elfström et al. (2014) suggested that factors other than food shortages, namely the avoidance of other bears or lack of experience with humans, explain bear incidences near settlements. On the Shiretoko Peninsula, the habituation of maternal bears to humans enhances the likelihood of human–bear conflict, especially in young males in the process of dispersal (Shimozuru, Shirane, Yamanaka, et al., 2020). Therefore, it would be premature to state that food shortages alone cause human–bear conflicts in this brown bear population. The fact that this study was able to reveal variation in the diets and body conditions of brown bears in the Rusha area, far from residential areas, is advantageous for understanding the natural ecology of brown bears. In future studies, we hope to clarify the characteristics of bears that appear in human settlements by comparing the diets and body conditions of brown bears around residential areas with the results of this study.

This study showed that coastal and subalpine foods are important for brown bear survival, in terms of both determining body condition in late summer and accelerating the risk of human‐caused mortality. Bear vertical movements between coastal and subalpine regions, revealed by scat content analysis, may contribute to forest ecosystems through seed dispersal (Shakeri et al. 2018; Willson & Gende, 2004) and the transport of marine‐derived nutrients (Drake et al. 2002; Helfield & Naiman, 2006; Hilderbrand et al. 1999). On the other hand, we cannot ignore the vulnerability of Shiretoko brown bears that depend on marine ecosystems and alpine vegetation, two of the habitats most strongly affected by climate change (Hoegh‐Guldberg & Bruno, 2010; Inouye, 2020). In Hokkaido, many factors that imply current effects of climate change on ecosystems have been observed, such as decreased seasonal sea ice (Makino & Sakurai, 2012) and reduction in the body size and population of salmon (Kaeriyama, 2008; Kishi et al., 2010). Although a phenological shift leading to the earlier flowering of alpine plants (Kudo & Hirao, 2006; Ogawa‐Onishi & Berry, 2013) may benefit brown bears awaiting the maturation of pine nuts in late summer, the predicted decreases in the habitat area of stone pine (Horikawa et al. 2009) will probably lead to greater changes in brown bear diet and behavior. These environmental changes will disrupt the vertical movements of brown bears and their benefits, resulting in negative impacts not only on the survival of brown bears but also on the entire forest ecosystem. In Yellowstone National Park, mountain pine beetle (*Dendroctonus ponderosae*) outbreaks promoted by warmer temperatures have caused mortality within about 82% of the whitebark pine stands (Macfarlane et al. 2013), resulting in a reduction in the digestible energy and protein content of the brown bear diet (López‐Alfaro et al. 2015). We must closely observe the Shiretoko Peninsula to see whether large‐scale environmental changes in the marine ecosystem and alpine vegetation will occur in the future.

In conclusion, we have revealed that subalpine pine nuts and coastal salmon, which are foods unique to the Shiretoko Peninsula, determine the summer body condition of female brown bears. In addition, August is the harshest season for brown bears on the peninsula, in particular when bears cannot heavily consume salmon. These findings may help to clarify the cause of human–bear conflict in Shiretoko, but it is still debatable whether food shortages and poor nutrition trigger the intrusion of bears into human residential areas. A previous study revealed that American black bears (*Ursus americanus*) use areas of higher human density in years when mast food production is poor in Colorado, USA (Baruch‐Mordo et al. 2014). By contrast, another study suggested that mast production does not determine brown bear movement behavior in Sweden (Hertel et al. 2019). The relationships among diet, body condition, and the behavioral patterns of bears need to be investigated further to establish effective management strategies for the mitigation of human–bear conflicts. In addition, females with offspring are particularly vulnerable to the adverse effects of food shortages in summer, which implies that significant declines in summer food resources may directly reduce foraging opportunities and negatively affect reproductive success. Our findings have important implications for predicting changes in reproductive success and behavioral patterns of brown bears with respect to annual fluctuation and even long‐term declines in the availability of coastal and subalpine foods.

## CONFLICT OF INTEREST

The authors have no conflict of interest to declare.

## AUTHOR CONTRIBUTIONS

**Yuri Shirane:** Conceptualization (lead); data curation (lead); formal analysis (lead); funding acquisition (lead); investigation (equal); methodology (lead); validation (equal); visualization (lead); writing – original draft (lead). **Mina Jimbo:** Formal analysis (supporting); investigation (equal); methodology (supporting); validation (equal); writing – review and editing (equal). **Masami Yamanaka:** Funding acquisition (lead); investigation (equal); project administration (lead); validation (equal); writing – review and editing (equal). **Masanao Nakanishi:** Investigation (equal); validation (equal); writing – review and editing (equal). **Fumihiko Mori:** Data curation (supporting); formal analysis (supporting); investigation (equal); methodology (supporting); validation (equal); writing – review and editing (equal). **Tsuyoshi Ishinazaka:** Funding acquisition (lead); supervision (supporting); writing – review and editing (equal). **Mariko Sashika:** Supervision (supporting); writing – review and editing (equal). **Toshio Tsubota:** Supervision (lead); writing – review and editing (equal). **Michito Shimozuru:** Funding acquisition (lead); investigation (equal); project administration (lead); supervision (lead); validation (equal); writing – review and editing (equal).

## Supporting information

Supplementary Material

Appendix S1

## Data Availability

Data used in this research are available in the supporting information.
