# Supplementary material for: Dining from the coast to the summit: Salmon and pine nuts determine the summer body condition of female brown bears on the Shiretoko Peninsula
Source: Ecol Evol. 2021 Mar 18;11(10):5204–19. doi: 10.1002/ece3.7410 (PMC8131783; doi:10.1002/ece3.7410)

**Appendix S1**

**Appendix A.** Correction factors, CF_D_ and CF_E_, used to calculate estimated dietary content and estimated digestible energy content for brown bear diet in the Rusha area of the Shiretoko Peninsula, eastern Hokkaido, Japan.

| Item | CF_D_ | Reference | CF_E_ | Reference |
| --- | --- | --- | --- | --- |
| Plants | 0.26 | Hewitt and Robinson 1996; Dahle et al. 1998; Persson et al. 2001 | 8.4 | Dahle et al. 1998; Persson et al. 2001 |
| Pine nuts |  |  |  |  |
| *Pinus* spp. | 1.54 | Hewitt and Robbins 1996 | 26.7 | Pritchard and Robbins 1990 |
| Drupes |  |  |  |  |
| *Prunus* spp. | 1.93 | Bojarska and Selva 2013 | 18.1 | Pritchard and Robbins 1990 |
| Berries |  |  | 18.1 | Pritchard and Robbins 1990 |
| *Vaccinium* spp. | 0.54 | Hewitt and Robbins 1996 |  |  |
| Other freshy fruits | 0.93 | Hewitt and Robbins 1996 |  |  |
| Acorns and other nuts | 1.54 | we chose to use the same correction factor as for pine nuts | 26.7 | we chose to use the same correction factor as for pine nuts |
| Mammals | - |  | 19.3 | Mealey 1980; Persson et al. 2001; Stenset et al. 2016 |
| Ungulate | 1.75 | Persson et al. 2001 | - |  |
| Large mammals | 2.00 | Persson et al. 2001 | - |  |
| Salmon |  |  |  |  |
| *Oncorhynchus* spp. | 40.80 | Hewitt and Robbins 1996 | 17.6 | Mealey 1980 |
| Insects | 1.10 | Hewitt and Robbins 1996 | - |  |
| Formicidae | - |  | 17.7 | Dahle et al. 1998; Persson et al. 2001; Ciucci et al. 2014 |
| Other insects | - |  | 11.3 | Dahle et al. 1998; Ciucci et al. 2014 |
| Other | - |  |  |  |
| Fungi | 0.26 | Stenset et al. 2016 | 10.0 | Dahle et al. 1998 |
| Birds | 1.50 | Dahle et al. 1998; Persson et al. 2001 | 18.8 | Dahle et al. 1998; Persson et al. 2001 |
| Shellfish | 1.10 | we chose to use the same correction factor as for Insects | 11.3 | we chose to use the same correction factor as for Insects |
| Amphipod | 1.10 | we chose to use the same correction factor as for Insects | 11.3 | we chose to use the same correction factor as for Insects |

**Appendix B.** Validation and correction of visual estimation in scat analysis

In this study, we collected scat samples during 2013–2018 and calculated EDC using results from the point-frame analysis; however, we did not bring scats back to the laboratory in 2012, so scat content data were only available as vFV in 2012. To investigate whether vFV estimated in 2012 could be compared to EDC estimated using the point-frame method in other years, we used data for scats collected in 2013–2018 to test the hypothesis that there would be no difference between vFV and EDC for any food category (*i.e.*, plants, pine nuts, drupes, berries, acorn and nuts, insects, mammals, and salmon) in any month within the same year. We tested this hypothesis using a linear regression with no intercept and excluded the category from the annual comparison when the *R*^2^ value was <0.70. If the *R*^2^ value was ≥0.70, we conducted a *t* test (*p* = 0.05) between the slope of the regression equation and a theoretical slope of 1.0 for complete correspondence. If the null hypothesis that regression coefficient = 1 was not rejected, we used vFV as equivalent to EDC. If the null hypothesis was rejected, we corrected vFV based on the regression equations. Then, we adjusted the “other” category so that the corrected EDC for each month totaled 100%. We conducted statistical analyses using R 4.0.2.

As a result, vFV was positively correlated with EDC calculated using the point-frame method (Appendix C). The *R*^2^ values obtained by regression analysis were more than 0.80 for all food items with the exception of digested traces of mammals (*R*^2^ = 0.12). The slope of the regression equation differed significantly from the theoretical slope of 1.0 for plants (*t*_28_ = 2.61, *p* = 0.015), drupes (*t*_28_ = 2.65, *p* = 0.013), berries (*t*_28_ = 4.80, *p* < 0.001), and pine nuts (*t*_28_ = 4.31, *p* <0.001) but did not differ for hard mast (*t*_28_ = 0.60, *p* = 0.554), salmon (*t*_28_ = 1.63, *p* = 0.114), and insects (*t*_28_ = 0.44, *p* = 0.664). Therefore, we used the vFV for hard mast, salmon, and insects as equivalent to EDC in the following analyses. In addition, we corrected vFV based on the regression equations for plants, drupes, berries, and pine nuts and used these values as equivalent to EDC in the following analyses. These categories were biased only in the same direction in most cases: plant, drupe, and berry categories were overestimated by visual estimation, and pine nut category was underestimated.

**Appendix C.** Equation for converting the percentage fecal volume estimated visually (vFV) in 2012 to estimated dietary content (EDC). This equation was based on linear regression with no intercept using vFV and EDC data for scats collected in 2013–2018.

| Categories | Regression equation | adjusted *R*^2^ | *p* value |
| --- | --- | --- | --- |
| Plants | *y* = 0.90*x* | 0.96 | *p* = 0.015 |
| Drupes | *y* = 0.87*x* | 0.92 | *p* =0.013 |
| Berries | *y* = 0.85*x* | 0.96 | *p* < 0.001 |
| Acorns and other nuts | *y* = 0.98*x* | 0.96 | *p* = 0.554 |
| Pine nuts | *y* = 1.15*x* | 0.97 | *p* < 0.001 |
| Mammals | *y* = 0.40*x* | 0.12 | *p* < 0.001 |
| Salmon | *y* = 1.10*x* | 0.93 | *p* = 0.114 |
| Insects | *y* = 0.96*x* | 0.73 | *p* = 0.664 |

**Appendix D.** Definition of grades (scores) used for each photograph attribute to select photographs for assessing body condition. This table was created based on a table in Shirane *et al*. 2020.

| Attribute | Score 1 (good) | | Score 2 (medium) | Score 3 (poor) | |
| --- | --- | --- | --- | --- | --- |
| **Photographing condition** | | | | | |
| (A) Camera focus | The picture is sharp with the contour of the bear’s body clearly visible. | The picture is blurry, but still clear enough to make out the contour of the bear's body. | | | The picture is too blurry to make out the contour of the bear's body. |
| (B) Camera tilt | The dorsal and ventral suface of the bear are vertical to the camera. | The dorsal and ventral suface of the bear deviate slightly from the vertical with the camera. | | | The dorsal and ventral suface of the bear deviates significantly from the vertical with the camera. |
| (C) Body angle | The body axis of the bear is perpendicular to the camera. | The body axis of the bear is angled slightly in the parallel plane, either back or forth. | | | The body axis of the bear is angled significantly in the parallel plane, either back or forth. |
| (D) Torso height measurability | Both the highest part of the waist and the lowest part of the abdomen are clearly visible. | The highest part of the waist or the lowest part of the abdomen is unclear or partly obscured, but can still be approximated. | | | The highest part of the waist and/or the lowest part of the abdomen are not visible due to another animal or object. |
| (E) Body/torso length measurability | The tip of nose (for HBL, EBL and PBL), the highest part of the shoulder (for PBL and HTL), and the base of tail are clearly visible. | The tip of nose, the highest part of the shoulder, or the base of tail is unclear or partly obscured, but can still be approximated. | | | The tip of nose, the highest part of the shoulder, and/or the base of tail are not visible due to another animal or object. |
| **Bear posture** | | | | | |
| (A) Body arch | No visible arching of the body. The bear stands parallel to the ground. | - | | | The legs or the central part of the body is significantly lifted or dropped. |
| (B) Body straightness | Right/left forelimb and hindlimb are in front of the other. The hip joint is not fully extended. | Right/left forelimb and hindlimb are inside the body or either of the legs is fully extended. | | | - |

**Appendix E.** Seasonal variation in the estimated dietary content (EDC) of 1,764 brown bear scat samples collected in the Rusha area of the Shiretoko Peninsula, Hokkaido, Japan during 2013–2018.


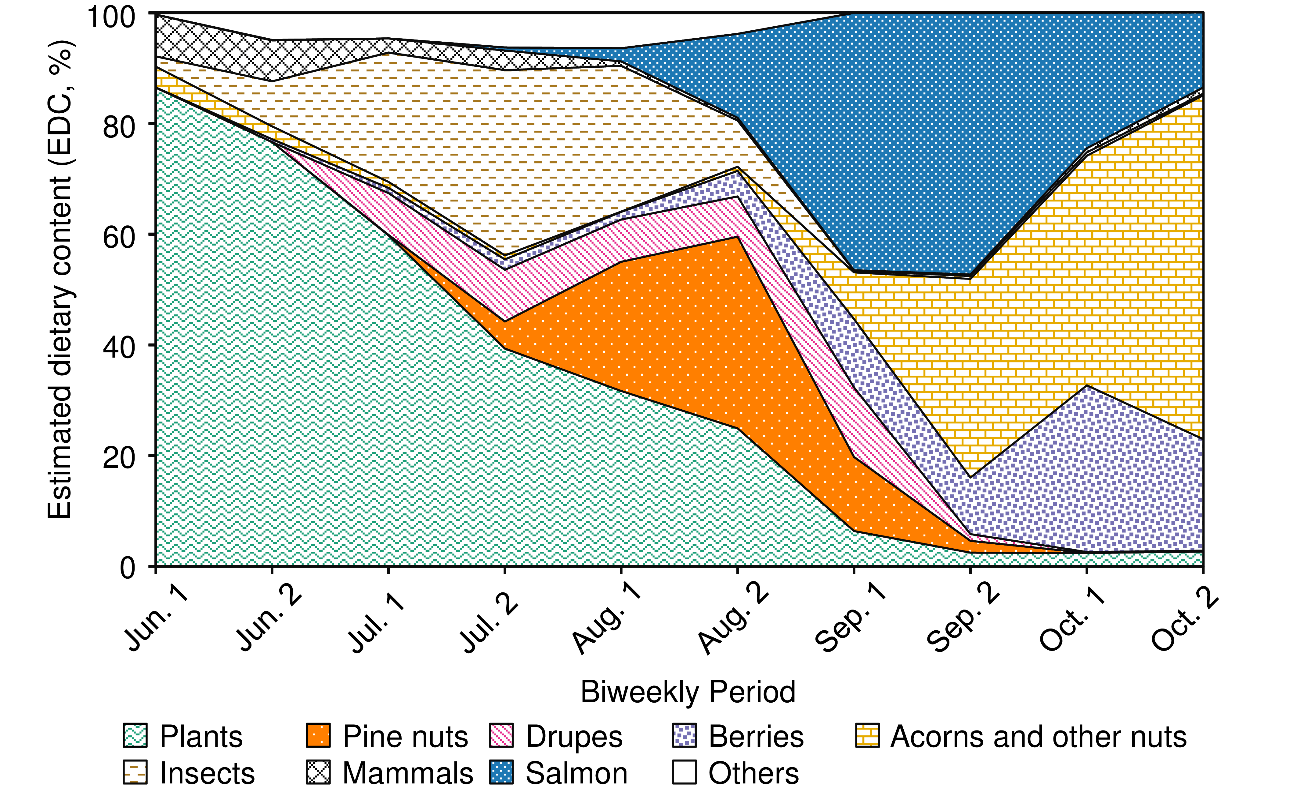


**Appendix F.** Examples of morphometric measurements and body condition evaluation (TH:HTL) using photographs of an adult female brown bear (bear ID: HC). These photographs were taken on (a) July 27, (b) August 23, and (c) October 7, 2015.

**
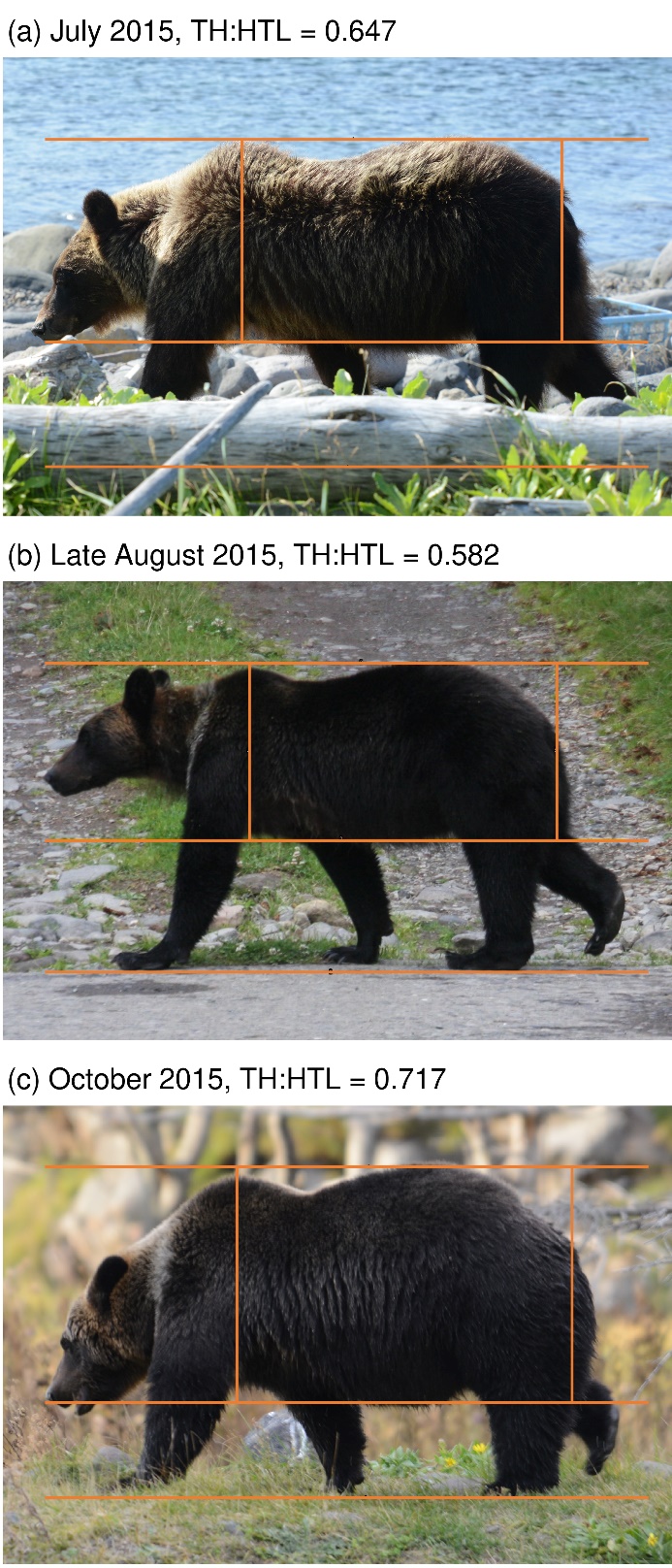
**

**Appendix G.** The standard diagnostic plots for checking the fitting procedures of generalized additive mixed model (the top-ranked model). (a) The normal Quantiles-Quantiles plot is very close to a straight line, suggesting reasonable distributional assumption. (b) The plot of residuals against linear predictor indicates that variance is approximately constant as the mean increases. (c) The histogram of residuals appears consistent with normality. (d) The plot of response against fitted values shows a positive linear relation with a good deal of scatter: nothing problematic.


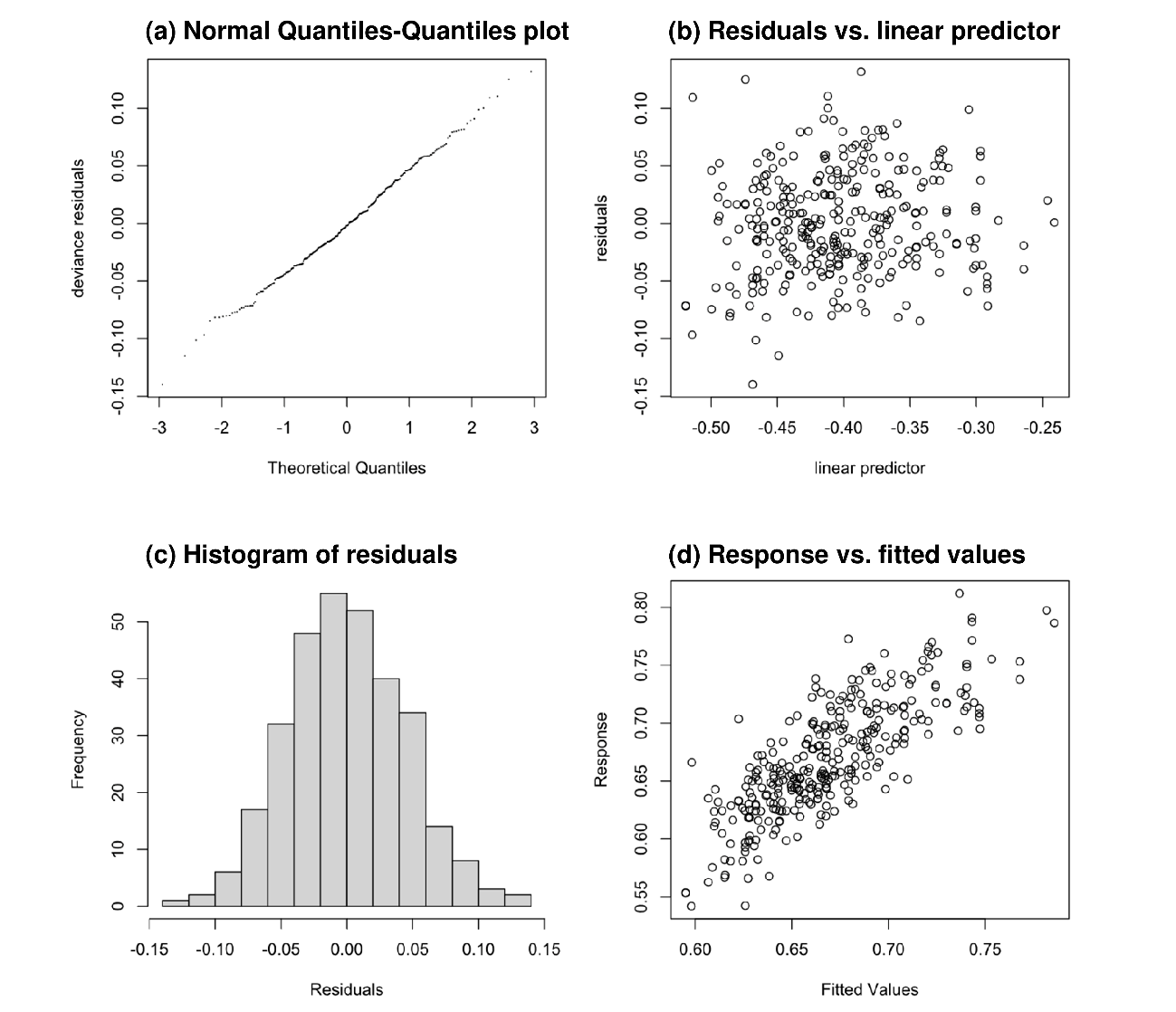


**Appendix H.** The standard diagnostic plots for checking the fitting procedures of generalized additive mixed model (the second-ranked model). (a) The normal Quantiles-Quantiles plot is very close to a straight line, suggesting reasonable distributional assumption. (b) The plot of residuals against linear predictor indicates that variance is approximately constant as the mean increases. (c) The histogram of residuals appears consistent with normality. (d) The plot of response against fitted values shows a positive linear relation with a good deal of scatter: nothing problematic.


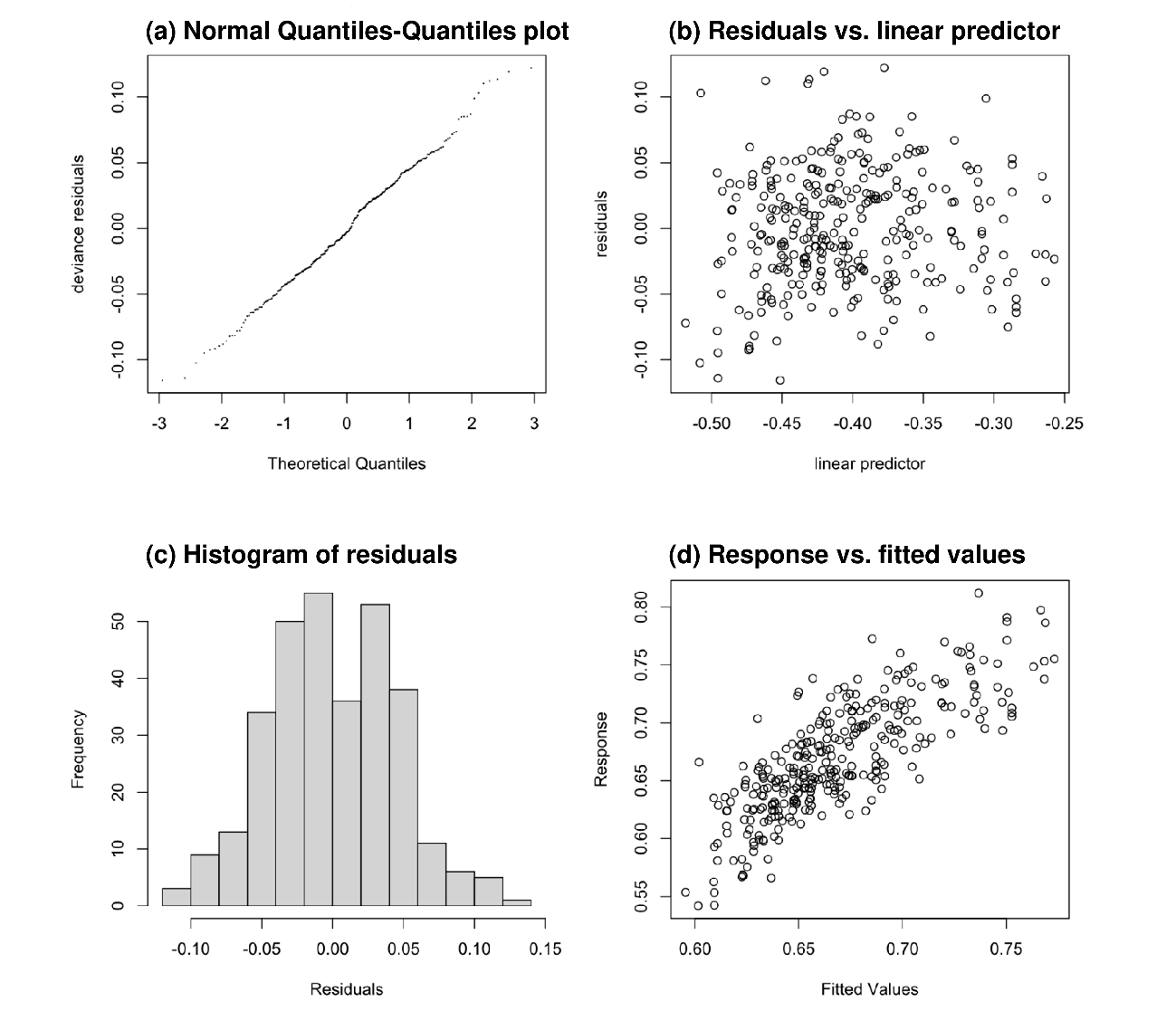

Supplement: Supplementary file 2 — Appendix S1 [file ECE3-11-5204-s002.docx]
